# AFF1 and AFF4 differentially regulate the osteogenic differentiation of human MSCs

**DOI:** 10.1038/boneres.2017.44

**Published:** 2017-09-26

**Authors:** Chen-chen Zhou, Qiu-chan Xiong, Xin-xing Zhu, Wen Du, Peng Deng, Xiao-bing Li, Yi-zhou Jiang, Shu-juan Zou, Cun-yu Wang, Quan Yuan

**Affiliations:** 1 State Key Laboratory of Oral Diseases & National Clinical Research Center for Oral Diseases, West China Hospital of Stomatology, Sichuan University, Chengdu, China; 2 Institute for Advanced Study, Shenzhen University, Shenzhen, China; 3 Laboratory of Molecular Signaling, Division of Oral Biology and Medicine, School of Dentistry and Broad Stem Cell Research Center, UCLA, Los Angeles, CA, USA; 4 Department of Orthodontics, West China Hospital of Stomatology, Sichuan University, Chengdu, China; 5 Department of Oral Implantology, West China Hospital of Stomatology, Sichuan University, Chengdu, China

## Abstract

AFF1 and AFF4 belong to the AFF (AF4/FMR2) family of proteins, which function as scaffolding proteins linking two different transcription elongation factors, positive elongation factor b (P-TEFb) and ELL1/2, in super elongation complexes (SECs). Both AFF1 and AFF4 regulate gene transcription through elongation and chromatin remodeling. However, their function in the osteogenic differentiation of mesenchymal stem cells (MSCs) is unknown. In this study, we show that small interfering RNA (siRNA)-mediated depletion of AFF1 in human MSCs leads to increased alkaline phosphatase (ALP) activity, enhanced mineralization and upregulated expression of osteogenic-related genes. On the contrary, depletion of AFF4 significantly inhibits the osteogenic potential of MSCs. In addition, we confirm that overexpression of AFF1 and AFF4 differentially affects osteogenic differentiation *in vitro* and MSC-mediated bone formation *in vivo*. Mechanistically, we find that AFF1 regulates the expression of DKK1 via binding to its promoter region. Depletion of DKK1 in HA-AFF1-overexpressing MSCs abrogates the impairment of osteogenic differentiation. Moreover, we detect that AFF4 is enriched in the promoter region of *ID1*. AFF4 knockdown blunts the BRE luciferase activity, *SP7* expression and ALP activity induced by BMP2 treatment. In conclusion, our data indicate that AFF1 and AFF4 differentially regulate the osteogenic differentiation of human MSCs.

## Introduction

Mesenchymal stem cells (MSCs) are pluripotent stem cells that can differentiate into osteoblastic, chondrogenic, and adipogenic lineages.^1,2^ The bone marrow is the main source of stem cells for mesenchymal tissue.^1^ Apart from that, MSCs can also be found in many other parts of the human body, including adipose tissue, chorionic villi of the placenta, amniotic fluid, peripheral blood, fetal liver, lung, and teeth.^3–6^

The osteogenic differentiation of MSCs is a complex process involving numerous signal molecules, including key transcription factors such as runt-related transcription factor 2 (Runx2) and Osterix, as well as various hormones.^7–11^ In addition, the Wnt/β-catenin, bone morphogenetic protein (BMP) and transforming growth factor-β (TGF-β) signaling pathways are indispensable during the osteogenic process.^12–15^ Recently, accumulating evidence has shown that epigenetic regulation plays a pivotal role in the osteogenic differentiation of MSCs.^16–18^ DNA methylation and histone modifications are the major mammalian epigenetic mechanisms involved in the progression from MSCs into terminally differentiated cells.^16,19–21^ For example, the expression level of histone deacetylase 1 (HDAC1) is decreased during osteoblast differentiation.^21^

Both AFF1 and AFF4 belong to the AFF (AF4/FMR2) family and regulate gene transcription epigenetically through elongation and chromatin remodeling.^22–24^ They share three conserved domains: an N-terminal homology domain, an AF4/lymphoid nuclear protein domain, and a C-terminal homology domain.^25^ Both function as scaffolding proteins linking two different transcription elongation factors, positive elongation factor b (P-TEFb) and ELL1/2, in super elongation complexes (SECs).^26,27^ Studies have shown that AFF1 and AFF4 are associated with acute lymphoblastic leukemia and FRAXE mental retardation.^27–31^ AFF1 promotes CD133 transcription and leukemia cell survival in multiple cancer cell lines.^32^ AFF1 and AFF4 also play important roles in HIV transactivation and are closely associated with HIV-1 Tat.^33,34^ However, the role of AFF1 and AFF4 in MSC osteogenic differentiation is largely unknown.

Although AFF1 and AFF4 are members of the same protein family with common structures and functions, we show that they differentially regulate the osteogenic differentiation of human MSCs *in vitro* and MSC-mediated bone formation *in vivo*. Mechanically, AFF1 controls the transcription of Dickkopf-related protein 1 (DKK1), while AFF4 is required for DNA-binding protein inhibitor ID1 transcription and BMP2-induced responses.

## Materials and methods

### Cell culture

Human bone marrow-derived mesenchymal stem cells (MSCs) were obtained from the American Type Culture Collection (ATCC, Manassas, VA, USA). The cells were cultured in Dulbecco’s modified Eagle’s medium (DMEM) supplemented with 10% fetal bovine serum (FBS, Gibco, Waltham, MA, USA), 100 U·mL^−1^ penicillin and 100 mg·mL^−1^ streptomycin (Gibco) at 37 °C in a humidified atmosphere of 5% CO_2_. To induce osteogenic differentiation, the cells were treated with osteogenic medium containing 50 μmol·L^−1^ ascorbic acid, 10 mmol·L^−1^ β-glycerophosphate, and 10 nmol·L^−1^ dexamethasone (all from Sigma, St. Louis, MO, USA).^35^ All experimental protocols and procedures were approved by the State Key Laboratory of Oral Diseases, West China Hospital of Stomatology, Sichuan University.

### Gene knockdown and overexpression

We obtained targeted and control small interfering RNAs (siRNAs) from Santa Cruz Biotechnology (Santa Cruz, Dallas, TX, USA), and transfections were performed using Lipofectamine RNAiMAX reagent (Invitrogen) according to the manufacturer’s instructions. The knockdown efficiency was verified by quantitative reverse transcription PCR (RT-PCR) and western blot 2 days after transfection.

For overexpression, lentiviruses expressing HA-AFF1, HA-AFF4 or empty vectors were purchased from GeneCopoeia (Guangzhou, China). MSCs were infected with these viruses in the presence of Polybrene (Sigma) for 24 h and selected by treatment with 1 μg·mL^−1^ puromycin (Sigma). The infection efficiency in the selected stable cells was confirmed by quantitative reverse transcription PCR (RT-PCR) and western blot.

### RNA isolation and quantitative RT-PCR

Total RNA was isolated using TRIzol reagent (Invitrogen, Waltham, MA, USA) according to the manufacturer’s instructions. cDNA was prepared from 1 μg aliquots of RNA using a PrimeScript RT reagent kit (Takara, Dalian, China). Quantitative real-time PCR was performed using SYBR Premix Ex Taq (Takara) with an ABI 7500 real-time PCR system (Applied Biosystems, Foster City, CA, USA).^36^ Relative expression levels were calculated using the 2^−ΔΔCt^ method by normalization to the expression of the *Gadph* housekeeping gene and were presented as the fold increase relative to the control.^9^

### Western blot

MSCs were lysed on ice in lysis buffer containing 50 mmol·L^−1^ Tris-HCl, 150 mmol·L^−1^ NaCl, 1 mmol·L^−1^ EDTA, 1% Nonidet P-40, and a protease inhibitor cocktail (Roche, Indianapolis, IN, USA). The cells were then centrifuged at 18 000×*g* for 15 min at 4 °C to remove the cell debris. The supernatants were heated at 95 °C for 5 min in sample buffer containing 2% SDS and 1% 2-mercaptoethanol, separated on 10% SDS–polyacrylamide gels, and transferred to PVDF membranes using a wet transfer apparatus (Bio-Rad, Hercules, CA, USA). The membranes were blocked with 5% milk for 1 h and then incubated with rabbit anti-AFF1 (Bethyl, Montgomery, TX, USA, 1:1 000), rabbit anti-AFF4 (Abcam, Cambridge, MA, USA, 1:1 000), rabbit anti-DKK1 (Cell Signaling, Danvers, MA, USA, 1:1 000), rabbit anti-ID1 (Abcam, 1:1 000), or mouse anti-α-tubulin (Sigma, 1:5 000) overnight, followed by a horseradish peroxidase-conjugated anti-rabbit or anti-mouse IgG (Jackson ImmunoResearch, West Grove, PA, USA). The antibody–antigen complexes were visualized with SuperSignal reagents (Pierce, Rockford, IL, USA).

### Cell proliferation assay

The proliferation of MSCs was analyzed using Cell Proliferation Reagent WST-1 (Roche). Briefly, 10 μL of reagent was added to each well, including five wells containing only medium for background subtraction. After a 1-hour incubation at 37 °C, the absorbance at 450 nm was measured using a Varioskan Flash microplate reader (Thermo Scientific, Waltham, MA, USA).

### ALP staining and activity

For ALP staining, cells were seeded in 24-well culture plates. After reaching confluence, the cells were incubated with osteogenic differentiation medium for 7 days. The cells were then fixed in 70% ethanol and incubated with a staining solution of 0.25% naphthol AS-BI phosphate and 0.75% Fast Blue BB dissolved in 0.1 mol·L^−1^ Tris buffer (pH 9.3). The ALP activity was quantified using a commercial kit according to the manufacturer’s protocol (Cell Biolab, San Diego, CA, USA) and normalized to the total protein levels.^36^

### Alizarin red staining

MSCs were cultured in osteogenic medium for 2–3 weeks, fixed with 10% neutral formalin for 5 min, and stained with 2% Alizarin red S (pH 4.2, Sigma) for 10 min.^37^ Mineralized bone nodules stained with Alizarin red were destained with 10% cetylpyridinium chloride in 10 mmol·L^−1^ sodium phosphate (pH 7.0), and the calcium concentration was determined by measuring the absorbance at 562 nm.^36^

### MSC transplantation

Three-month-old immunocompromised beige mice were obtained from the Experimental Animal Center of the university and housed in pathogen-free facilities under a 12-h light and 12-h dark cycle. All procedures were conducted in accordance with *The Guidelines for the Care and Use of Laboratory Animals* of the State Key Laboratory of Oral Diseases, West China Hospital of Stomatology, Sichuan University. Approximately 5×10^6^ cells were mixed with 60 mg of pure phase beta-tricalcium phosphate particles (SynthoGraft, Bicon, Boston, MA) and then transplanted subcutaneously under the dorsal surface as previously described.^36^ The samples were collected 3 and 6 weeks after transplantation and decalcified with 10% EDTA. Paraffin sections were generated and stained with hematoxylin and eosin.

### Chromatin immunoprecipitation assay

The chromatin immunoprecipitation (ChIP) assay was performed using a Simple ChIP Assay Kit (Cell Signaling Technology) according to the manufacturer’s protocol with an antibody against AFF1 (Bethyl), AFF4 (Abcam) or the control normal rabbit IgG (Santa Cruz Biotechnology). After dissociating the DNA-protein complexes, the pulled-down DNA and input DNA were subject to quantitative RT-PCR analysis with primers designed to amplify target promoter regions. The results were expressed as the percentage of input DNA.

### Luciferase assay

MSCs were transfected with AFF4 or control siRNA together with 100 ng of BRE luciferase (#45126 Addgene, Cambridge, MA, USA) and 50 ng of CMV-β-galactosidase constructs using Lipofectamine 2000 transfection reagent (Invitrogen). After approximately 24 h, the cells were starved in serum-free medium overnight followed by treatment with 100 ng·mL^−1^ BMP2 for 6 h. The luciferase and β-galactosidase activities of total cell lysates were determined using Luc-Screen and Galacto-Star kits (Applied Biosystems).

### Statistical analysis

All values are presented as the mean±s.e. A two-tailed Student’s *t*-test and one-way analysis of variance (ANOVA) followed by Tukey’s test were used for single comparisons and multiple comparisons to assess the statistical significance of the differences among each pair of data sets. A *P-*value <0.05 was considered to be statistically significant.

## Results

### Depletion of AFF1 improves the osteogenic differentiation of MSCs

**Figure 1:**
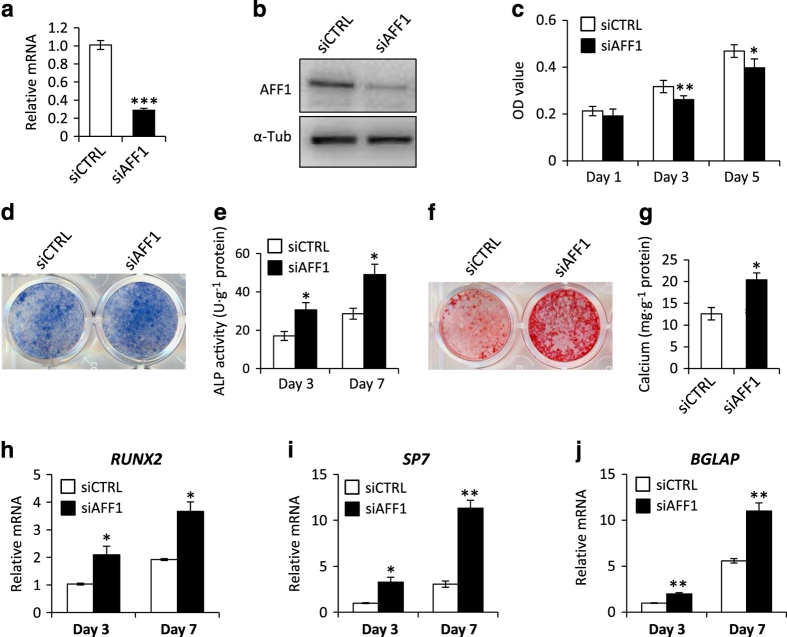
Depletion of AFF1 improves osteogenic differentiation. (**a**) Real-time RT-PCR shows successful knockdown of *AFF1. n*=3. ***P<0.001. (**b**) Western blot analysis of AFF1. (**c**) Depletion of AFF1 inhibits the proliferation of MSCs. *n*=5. *P<0.05 and **P<0.01. (**d**) Representative images of alkaline phosphatase (ALP) staining. Depletion of AFF1 leads to more intense staining. (**e**) Quantitative analyses of ALP activity. *n*=5. *P<0.05. (**f**) Representative images of Alizarin red S (ARS) staining of MSCs. (**g**) Quantitative analyses of calcium mineralization. *n*=5. *P<0.05. (**h**–**j**) Real-time RT-PCR reveals elevated mRNA expression of *RUNX2*, *SP7* and *BGLAP*. *n*=3. *P<0.05 and **P<0.01.

To investigate the role of AFF1 in osteogenic differentiation, we first depleted AFF1 in human MSCs using siRNA. The knockdown efficiency was confirmed by RT-PCR and western blot (Figure 1a and b). AFF1 depletion decreased the proliferation of MSCs (Figure 1c). After osteogenic induction for 7 days, we found that siRNA-mediated depletion of AFF1 led to more intense staining of alkaline phosphatase (ALP), an early marker of osteoblastic differentiation (Figure 1d). This observation was confirmed by quantitatively measuring ALP activity on days 3 and 7 (Figure 1e). We also assessed extracellular matrix (ECM) mineralization by Alizarin red S (ARS) staining. As shown in Figure 1f and g, mineralization was significantly increased after AFF1 depletion. In addition, AFF1 knockdown upregulated the expression of osteogenic-related genes, such as *Runx2*,* Osterix (SP7)*, and *Osteocalcin* (*BGLAP*) (Figure 1h–j).

### Overexpression of AFF1 impairs osteoblastic differentiation

**Figure 2:**
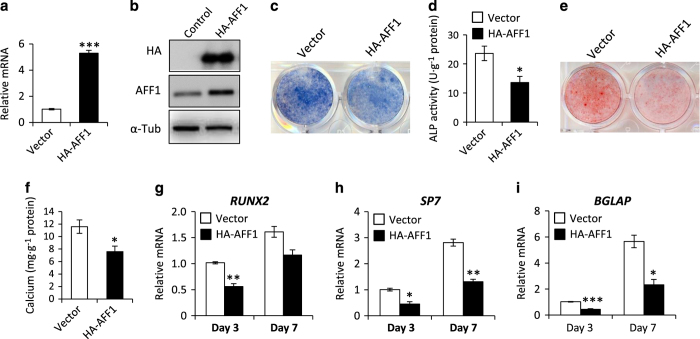
Overexpression of AFF1 impairs osteogenic differentiation. (**a**) Real-time RT-PCR shows that the mRNA level of *AFF1* is significantly increased*. n*=3. ***P<0.001. (**b**) Western blot analysis of AFF1. (**c**) Representative images of ALP staining. Overexpression of AFF1 decreases the intensity of the staining. (**d**) Quantitative analyses of ALP activity. *n*=5. *P<0.05. (**e**) Representative images of ARS staining of MSCs. (**f**) Quantitative analyses of calcium mineralization. *n*=5. *P<0.001. (**g**–**i**) Real-time RT-PCR reveals decreased mRNA expression of *RUNX2*, *SP7* and *BGLAP*. *n*=3. *P<0.05, **P<0.01 and ***P<0.001.

To confirm the role of AFF1 in osteoblastic differentiation, we investigated the effect of ectopic overexpression of AFF1 on osteoblastic differentiation. Human MSCs were stably transduced with lentiviral particles expressing HA-AFF1 (Figure 2a and b). We found that overexpression of AFF1 decreased the ALP activity and mineralization of MSCs (Figure 2c–f). In addition, the expression of osteogenic-related genes, such as *Runx2, SP7*, and *BGLAP,* was significantly repressed in AFF1-depleted cells subjected to osteogenic induction for 5 days (Figure 2g–i).

### Depletion of AFF4 decreases osteogenic differentiation

**Figure 3:**
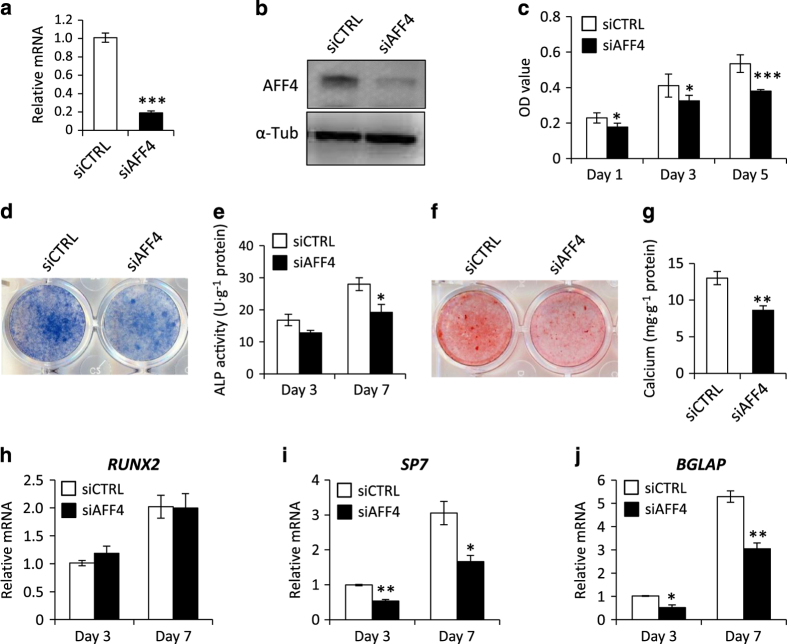
Depletion of AFF4 decreases osteogenic differentiation. (**a**) Real-time RT-PCR shows successful knockdown of *AFF4. n*=3. ***P<0.001. (**b**) Western blot analysis of AFF4. (**c**) Depletion of AFF4 reduces the proliferation of MSCs. *n*=5. *P<0.05 and ***P<0.001 (**d**) Representative images of ALP staining. Depletion of AFF4 decreases the intensity of the staining. (**e**) Quantitative analyses of ALP activity. *n*=5. *P<0.05. (**f**) Representative images of ARS staining of MSCs. (**g**) Quantitative analyses of calcium mineralization. *n*=5. **P<0.01. (**h**–**j**) Real-time RT-PCR reveals reduced mRNA expression of *SP7* and *BGLAP*. *n*=3. *P<0.05 and **P<0.01.

As both AFF4 and AFF1 are key members of the AFF family and share highly conserved functional domains involved in gene transcription regulation, including conserved N- and C-terminal domains, a serine-rich transactivation domain, and an ALF homology region,^25,38^ we sought to verify whether AFF4 plays a similar role as AFF1 in regulating the osteogenic differentiation of MSCs. AFF4 was knocked down in MSCs using siRNA (Figure 3a and b), resulting in reduced MSC proliferation (Figure 3c). Surprisingly, depletion of AFF4 significantly reduced the alkaline phosphatase (ALP) activity and extracellular matrix mineralization, indicating that it had an opposite effect on osteogenic differentiation compared with AFF1 (Figure 3d–g). In addition, knockdown of AFF4 inhibited the expression of osteogenic-related genes, such as *Runx2, SP7,* and *BGLAP* (Figure 3h–j).

### Overexpression of AFF4 enhances the osteoblastic differentiation of MSCs

**Figure 4:**
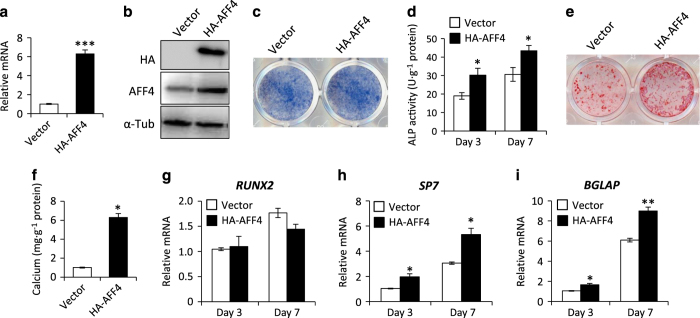
Overexpression of AFF4 enhances osteogenic differentiation. (**a**) Real-time RT-PCR shows that the mRNA level of *AFF4* is significantly increased*. n*=3. ***P<0.001. (**b**) Western blot analysis of AFF4. (**c**) Representative image of ALP staining. Overexpression of AFF4 leads to more intense staining. (**d**) Quantitative analyses of ALP activity. *n*=5. *P<0.05. (**e**) Representative images of ARS staining of MSCs. (**f**) Quantitative analyses of calcium mineralization. *n*=5. *P<0.001. (**g**–**i**) Real-time RT-PCR reveals increased mRNA expression of *SP7* and *BGLAP*. *n*=3. *P<0.05 and **P<0.01.

To investigate the effects of ectopic overexpression of AFF4 on osteoblastic differentiation, human MSCs were stably transduced with lentiviral particles expressing HA-AFF4 (Figure 4a and b). As expected, the ALP activity and mineralization of MSCs were markedly enhanced after AFF4 overexpression (Figure 4c–f). Moreover, an RT-PCR assay showed that the expression of the abovementioned osteogenic-related genes was significantly elevated after AFF4 overexpression and 5 days of osteogenic induction (Figure 4h–i).

### AFF1 and AFF4 differentially regulate MSC-mediated bone formation *in vivo*

**Figure 5:**
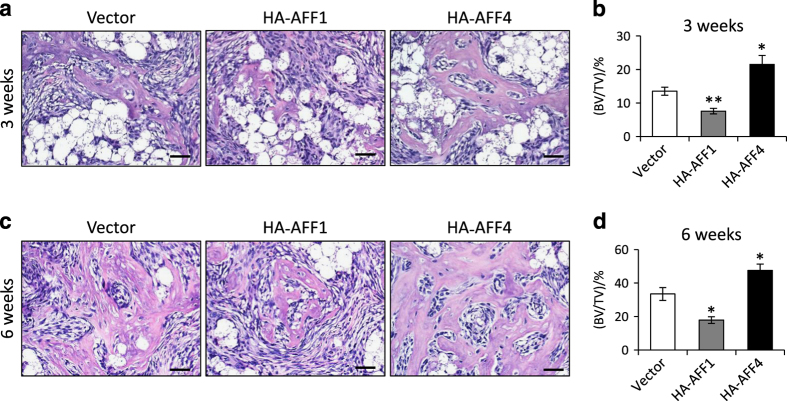
AFF1 and AFF4 differentially regulate bone formation *in vivo*. (**a**) Representative images of H&E staining of ectopic bone formation at 3 weeks. There is very little newly generated bone, while many β-TCP particles (the white bubble-like spots) remain. (**b**) Quantitative analyses of bone volume versus total tissue volume (BV/TV) at 3 weeks. *n*=5. *P<0.05 and **P<0.01. (**c**) Representative images of H&E staining at 6 weeks. Cells overexpressing HA-AFF1 form less bone tissue, while those overexpressing HA-AFF4 exhibit more bone formation. (**d**) BV/TV at 6 weeks. *n*=5. *P<0.05.

To verify our findings *in vitro*, we examined whether the overexpression of AFF1 and AFF4 differentially affected MSC-mediated bone formation *in vivo*. To this end, we subcutaneously implanted MSCs stably overexpressing HA-AFF1 or HA-AFF4 with β-TCP scaffolds into immunocompromised mice. After 3 weeks, H&E staining showed that there was very little newly generated bone, while many β-TCP particles remained (Figure 5a). However, the bone volume in the HA-AFF4 group was significantly higher than that in the control and HA-AFF1 groups (Figure 5a and b). At 6 weeks, much more bone was observed in all three groups compared with that at 3 weeks. Mice implanted with AFF1-overexpressing MSCs showed much less bone tissue (Figure 5c and d). By contrast, increased bone formation was observed in mice implanted with AFF4-overexpressing MSCs (Figure 5c and d), and bone marrow was observed.

### AFF1 controls DKK1 transcription

**Figure 6:**
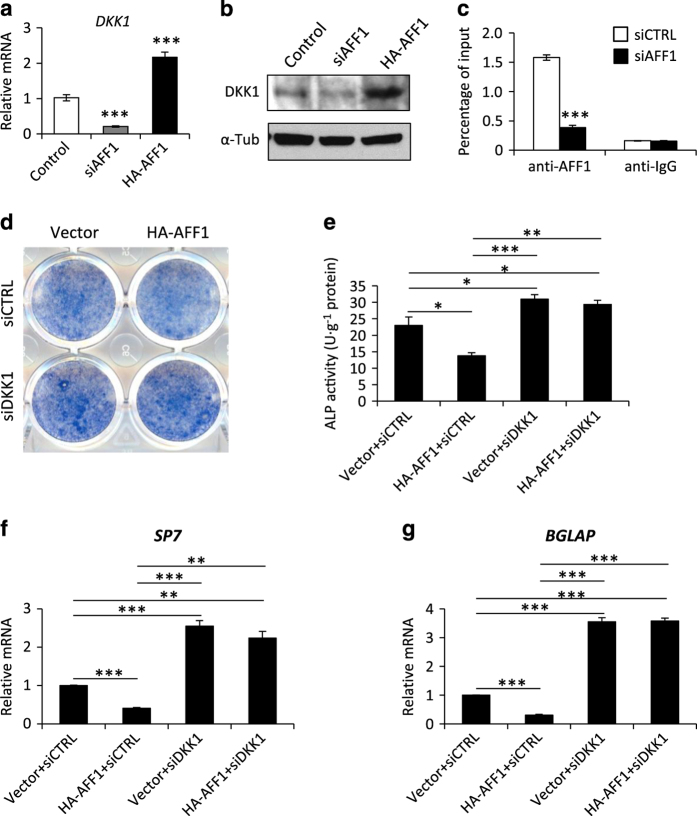
AFF1 controls DKK1 transcription. (**a**) Real-time RT-PCR shows altered expression of *DKK1. n*=3. ***P<0.001. (**b**) Western blot analysis of DKK1. (**c**) A ChIP assay for AFF1 shows that it binds to the promoter region of *DKK1*. *n*=4. ***P<0.001. (**d**) Representative images of ALP staining. (**e**) Quantitative analyses of ALP activity. *n*=5. *P<0.05, **P<0.01 and ***P<0.001. (**f**, **g**) Real-time RT-PCR of *SP7* and *BGLAP*. *n*=3. **P<0.01 and ***P<0.001.

DKK1 is an antagonistic inhibitor of the Wnt/β-catenin signaling pathway, which plays a critical role in skeletal development and osteogenesis.^14,39^ Therefore, we want to know whether AFF1, as an elongation factor, could mediate DKK1 gene transcription. Thus, we examined the expression of DKK1 after AFF1 depletion in MSCs. We found that depletion of AFF1 in MSCs significantly reduced the mRNA and protein levels of DKK1 (Figure 6a and b). Conversely, overexpression of AFF1 increased the expression of DKK1 (Figure 6a and b). In addition, we performed an anti-AFF1 ChIP assay, which demonstrated that AFF1 bound to the promoter region of *DKK1*. The ChIP signal for AFF1 was almost completely abolished by AFF1 knockdown (Figure 6c). These data suggested that AFF1 might control the osteoblastic differentiation of MSCs by regulating *DKK1* transcription.

To further elucidate the mechanism, we performed rescue experiments by knocking down DKK1 in stable HA-AFF1-expressing MSCs using siRNA. Depletion of DKK1 significantly abolished the inhibition of ALP activity triggered by AFF1 overexpression (Figure 6d and e) as well as the expression of *SP7*, a master transcription factor for osteogenic differentiation (Figure 6f), and GBLAP, a marker for mature osteoblasts (Figure 6g).

### AFF4 is required for ID1 transcription and BMP2-induced responses

**Figure 7:**
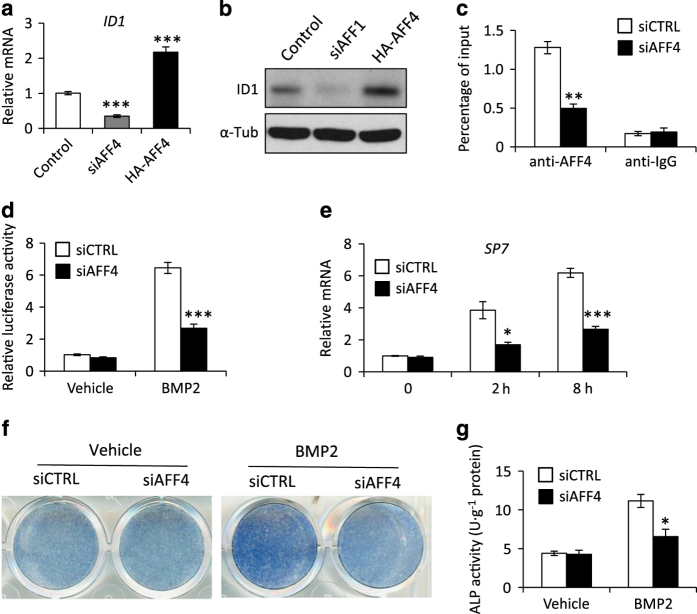
AFF4 is required for ID1 transcription and BMP2-induced responses. (**a**) Real-time RT-PCR shows altered expression of *ID1. n*=3. ***P<0.001. (**b**) Western blot analysis of ID1. (**c**) A ChIP assay for AFF4 shows that it binds to the promoter region of *ID1*. *n*=4. **P<0.01. (**d**) Relative luciferase activity after BMP2 (100 ng·mL^−1^) treatment for 6 h. Depletion of AFF4 blunts the BMP2-induced luciferase activity. *n*=4. ***P<0.001. (**e**) Real-time RT-PCR indicates that knockdown of AFF4 decreased *SP7* expression after BMP2 (100 ng·mL^−1^) treatment. *n*=3. *P<0.05 and ***P<0.001. (**f**) Representative images of ALP staining. (**g**) Quantitative analyses of ALP activity. Knockdown of AFF4 decreases the ALP activity induced by BMP2 treatment. *n*=5. *P<0.05.

The DNA-binding protein inhibitor ID1 is a target of the BMP pathway and plays an important role in regulating the differentiation of stem cells.^12,40^ Here, we found that depletion of AFF4 significantly reduced the mRNA level of ID1 as well as its protein levels (Figure 7a and b). By contrast, overexpression of AFF4 induced the expression of ID1 (Figure 7a and b). We then performed a ChIP assay and observed that AFF4 was enriched in the promoter region of ID1, suggesting that AFF4 might control osteogenic differentiation via the BMP pathway by regulating *ID1* transcription (Figure 7c). To this end, we performed a BRE luciferase assay and found that AFF4 depletion markedly restricted BMP2-induced responses (Figure 7d). As BMP2 induces the expression SP7, a master transcription factor for osteogenic differentiation, we examined whether this induction requires AFF4. Quantitative RT-PCR revealed that deletion of AFF4 blunted the expression of *SP7* induced by BMP2 treatment (Figure 7e). In addition, knockdown of AFF4 also decreased the ALP activity induced by BMP2 treatment (Figure 7f and g).

## Discussion

As a set of transcriptional regulators, AFF family proteins play important roles in numerous biological processes, including transcription regulation, chromatin remodeling and leukemia.^24,28,41^ Their relationship with leukemia and functions in HIV transcription has been widely investigated.^29,30,33,34^ Nevertheless, whether they function similarly in cellular differentiation, especially in the osteogenic differentiation of MSCs, remains unknown. In the present study, we show that AFF1 depletion enhances the expression of osteogenic-related genes, ALP activity and mineralization. Overexpression of AFF1 impairs osteogenic differentiation and MSC-mediated bone formation *in vivo*. Conversely, we find that AFF4, another member of the AFF family, has an opposite regulatory function in the expression of osteogenic-related genes, ALP activity and mineralization *in vitro* as well as in bone formation *in vivo*. These findings indicate that AFF1 and AFF4 differentially regulate the osteogenic differentiation of MSCs. The expression levels of AFF2 and 3 are too low to be detected in hMSCs (unpublished data).

AFF1 and AFF4 share some conserved functional domains in their protein structures.^24,41^ Both of them are reported to form fusion genes with the mixed lineage leukemia *(MLL)* gene, which is associated with leukemia.^32,42^ Both AFF1 and AFF4 act as partners of P-TEFb to regulate transcription elongation. Previous studies have shown that either AFF1 or AFF4 could participate in the formation of different SEC subtypes by serving as a scaffolding protein.^34^ Other researchers also found that AFF4 binds to Tat-P-TEFb, which indirectly stimulates recognition of the HIV promoter.^33^ In addition, both AFF1 and AFF4 are scaffolding proteins that link different parts of SECs.^28,34^ Despite these similarities, these two proteins have opposite effects on the osteogenic differentiation of MSCs. Overexpression of AFF1 impairs MSC-mediated bone formation, whereas overexpression of AFF4 enhances this process. Mechanistically, ChIP experiments suggest that both AFF1 and AFF4 could function as transcription factors to mediate the transcriptional activation of the key regulators in MSC osteogenic differentiation by binding to the promoter regions of these genes. AFF1, but not AFF4, affects differentiation by regulating *DKK1* transcription, while AFF4 regulates *ID1* transcription to control osteoblastic differentiation through the BMP pathway. These findings are consistent with a previous report that demonstrated that genes regulated by SECs containing AFF1 or AFF4 are largely non-overlapping.^43^

On the basis on previous studies and the findings of this study, we speculate that there are several potential reasons for the different effects of AFF1 and AFF4 on the osteoblastic differentiation of MSCs. It is well known that SECs regulate gene expression at the transcriptional level.^38^ Although AFF1 and AFF4 are components of SECs, they may be independently localized and are not found together in a single SEC.^27,43^ These two proteins may also form a heterodimer, which is not necessary in SEC assembly.^43^ A previous study showed that the subnuclear distribution of AFF1 is diffuse, while AFF4 is mainly found on SC35 in nuclear speckles.^28^ Furthermore, the gene targets of AFF1 and AFF4 are largely distinct.^43^ Taken together, our data further demonstrated that these two proteins function differently during osteogenesis by regulating different signal pathways.

In summary, we show that AFF1 and AFF4 differentially regulate the osteogenic differentiation of human MSCs *in vitro* and MSC-mediated bone formation *in vivo*. Mechanically, AFF1 controls the transcription of DDK1, while AFF4 is required for ID1 transcription and BMP2-induced responses. Our data indicate that AFF1 and AFF4 are critical epigenetic regulators of the osteogenic differentiation of human MSCs.
