# Correction to: AFF1 and AFF4 differentially regulate the osteogenic differentiation of human MSCs

**DOI:** 10.1038/s41413-020-0093-4

**Published:** 2020-03-31

**Authors:** Chen-Chen Zhou, Qiu-Chan Xiong, Xin-Xing Zhu, Wen Du, Peng Deng, Xiao-Bing Li, Yi-Zhou Jiang, Shu-Juan Zou, Cun-Yu Wang, Quan Yuan

**Affiliations:** 1 State Key Laboratory of Oral Diseases & National Clinical Research Center for Oral Diseases, West China Hospital of Stomatology, Sichuan University, Chengdu, China; 2 Institute for Advanced Study, Shenzhen University, Shenzhen, China; 3 Laboratory of Molecular Signaling, Division of Oral Biology and Medicine, School of Dentistry and Broad Stem Cell Research Center, UCLA, Los Angeles, CA USA; 4 Department of Orthodontics, West China Hospital of Stomatology, Sichuan University, Chengdu, China; 5 Department of Oral Implantology, West China Hospital of Stomatology, Sichuan University, Chengdu, China

Correction to: *Bone Research* 10.1038/boneres.2017.44, published online 26 September 2017

During a reread of our article previously published in Bone Research, we regrettably found that the second lane of originally published Fig. 7b was labeled incorrectly. It should be siAFF4. And in Figs. 6b and Fig. 7b, the images of a-tublin were inadvertently misused. The correct images have been replaced. We sincerely apologize for this oversight, but it does not affect any of our original conclusions.

**Figure:**
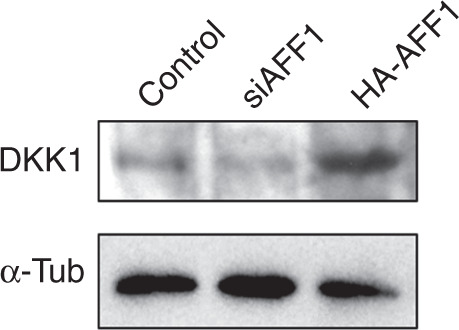
Fig. 6

**Figure:**
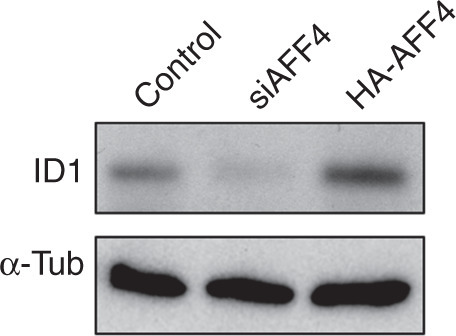
Fig. 7

